# Energy expenditure in critically ill patients estimated by population-based equations, indirect calorimetry and CO_2_-based indirect calorimetry

**DOI:** 10.1186/s13613-016-0118-8

**Published:** 2016-02-18

**Authors:** Mark Lillelund Rousing, Mie Hviid Hahn-Pedersen, Steen Andreassen, Ulrike Pielmeier, Jean-Charles Preiser

**Affiliations:** Center for Model-based Medical Decision Support (MMDS), Department of Health Science and Technology, Aalborg University, Fredrik Bajers Vej 7E, 9220 Aalborg East, Denmark; Department of Intensive Care, Erasme University Hospital, Université Libre de Bruxelles, 808 Route de Lennik, 1070 Brussels, Belgium

**Keywords:** Energy expenditure, Metabolic rate, Caloric intake, Nutritional support, Critically ill, Indirect calorimetry, Respiratory quotient, VCO_2_

## Abstract

**Background:**

Indirect calorimetry (IC) is the reference method for measurement of energy expenditure (EE) in mechanically ventilated critically ill patients. When IC is unavailable, EE can be calculated by predictive equations or by VCO_2_-based calorimetry. This study compares the bias, quality and accuracy of these methods.

**Methods:**

EE was determined by IC over a 30-min period in patients from a mixed medical/postsurgical intensive care unit and compared to seven predictive equations and to VCO_2_-based calorimetry. The bias was described by the mean difference between predicted EE and IC, the quality by the root mean square error (RMSE) of the difference and the accuracy by the number of patients with estimates within 10 % of IC. Errors of VCO_2_-based calorimetry due to choice of respiratory quotient (RQ) were determined by a sensitivity analysis, and errors due to fluctuations in ventilation were explored by a qualitative analysis.

**Results:**

In 18 patients (mean age 61 ± 17 years, five women), EE averaged 2347 kcal/day. All predictive equations were accurate in less than 50 % of the patients with an RMSE ≥ 15 %. VCO_2_-based calorimetry was accurate in 89 % of patients, significantly better than all predictive equations, and remained better for any choice of RQ within published range (0.76–0.89). Errors due to fluctuations in ventilation are about equal in IC and VCO_2_-based calorimetry, and filtering reduced these errors.

**Conclusions:**

This study confirmed the inaccuracy of predictive equations and established VCO_2_-based calorimetry as a more accurate alternative. Both IC and VCO_2_-based calorimetry are sensitive to fluctuations in respiration.

## Background

The determination of energy expenditure (EE) can help clinicians to prescribe caloric intake during the late phase of critical illness, particularly in obese, cachectic or burned patients [1]. The reference method to determine EE is indirect calorimetry (IC) [2], which uses the Weir equation [3] to provide an estimate of EE from measured oxygen consumption (VO_2_) and carbon dioxide production (VCO_2_). However, the use of IC is limited by the associated costs, necessary training and demand on resources (e.g., time, equipment and staff) [4, 5]. Furthermore, IC measurements may not be feasible because of logistic or technical difficulties, in about 35–40 % of patients even under conditions of a clinical prospective trial [6, 7].

Regardless of the nutritional target, relative to EE, for a patient, EE should be accurately determined. The use of EE determined by predictive equations is recommended when IC cannot be used. For instance, the American College of Chest Physicians (ACCP) equation [8] uses body mass (BM) as the only variable describing the patient: EE(ACCP) = (25–30 kcal/kg/day · BM). European [9] and Canadian [10] guidelines concur and both recommend a target of 20–25 kcal/kg/day. Other equations also use the patient’s height and age and gender (Harris–Benedict [11] and Mifflin St Jeor [12]). The Penn State equations [13, 14] add respiratory minute volume (MV) and body temperature to further describe the state of the patient.

Reviews by Tatucu-Babet et al. [6] and Frankenfield et al. [15] of the extensive body of the literature on predictive equations conclude that they often are inaccurate. Both reviews used a ±10 % difference between the predictive equations and IC to assess over- or underestimations of EE. Frankenfield et al. [15] found that the four equations reviewed all had over- and/or underestimations larger than 10 % in at least 18 % of the patients. Tatucu-Babet et al. [6] found that 12 % of the reviewed predictive equations on average over the patient group studied overestimated EE by more than 10 % and up to 66 % in individual patients. Underestimation was even more frequent with 38 % of the equations underestimating EE by more than 10 % and up to 41 % in individual patients. The frequent underestimations were partially compensated for by multiplying the EE estimated by the predictive equations by a stress factor (SF) and most of the studies evaluating the Harris–Benedict equation used a SF, which ranged from 1.13 to 1.6. This large range of SF may partially be due to interpatient differences, but also to systematic variations of SF due to the severity and type (sepsis, trauma/surgery, burns) of insult [16–18] as well as the time elapsed since the insult [16, 17]. The value of SF is therefore cohort specific, depending on both patient mix and other clinical circumstances.

An alternative may be “VCO_2_-based calorimetry” where EE is calculated only from VCO_2_, routinely measured by capnometers connected to the ventilatory circuit in mechanically ventilated patients [19]. In this paper, we investigate a method to calculate the VCO_2_-based EE from a modified Weir equation [3]: EE(VCO_2_) = ((5.5 min/ml · RQ^−1^ + 1.76 min/ml) · VCO_2_ − 26)kcal/day [20]. In a clinical application of VCO_2_-based calorimetry where VO_2_ is not measured, the respiratory quotient (RQ) for the individual patient is unknown and a value of RQ for the individual patient must therefore be chosen. This value may be set to the average from a patient cohort [20, 21] or can be individualized by calculating it from the patient’s nutrition [22, 23]. The purpose of this study is to determine the accuracy of VCO_2_-based calorimetry using the modified Weir equation stated above compared with the accuracy of commonly used predictive equations for EE, using IC as the reference method. In clinical practice, the VCO_2_ measurements are presumably taken using the ventilator’s capnometer. The scope of this paper is not the potential discrepancy between VCO_2_ measurements from capnometers in metabolic monitors and in ventilators, but only the accuracy of the VCO_2_-based calorimetry compared with IC. Possible sources of error in the VCO_2_-based calorimetry and IC will be assessed by a qualitative analysis of data, including a sensitivity analysis of the choice of RQ value.

## Methods

### Patients

An observational trial was conducted at a mixed medical/postsurgical intensive care unit (ICU) at Erasme University Hospital of Brussels, Belgium. No ethics committee approval was necessary as only noninvasive and anonymized data were collected. Eighteen patients 18 years or older were included as soon as possible after ICU admission, if they were intubated and mechanically ventilated. Height, gender, body mass, temperature, diagnosis, mode of ventilation, APACHE 2 score at admission [24], and sedation were recorded. VO_2_, VCO_2_, end-tidal CO_2_ (ET-CO_2_), FiO_2_, MV and RQ were measured over a 30-min period. The metabolic monitor used was a Compact Airway Module, E-CAiOVX, mounted in a Compact Anesthesia Monitor (GE Healthcare, Little Chalfont, Buckinghamshire, UK), which offers continual VCO_2_ and VO_2_ measurements [25]. The Compact Airway Module determines VCO_2_ and VO_2_ within ±10 % when FiO_2_ < 65 % [26].

EE is determined, using the Weir Eq. (3):

1 $${\text{EE}}\left( {\text{IC}} \right) = \left( {5.5\;{\text{min/ml}} \cdot {\text{VO}}_{2} + 1.76\;{\text{min/ml}} \cdot {\text{VCO}}_{2} - 1.99\;{\text{day/g}} \cdot {\text{N}}} \right) {\text{kcal/day}}$$

with a standard setting of *N* = 13 g/day [26], as ureic nitrogen was not measured in the study, yielding:

2 $${\text{EE}}\left( {\text{IC}} \right) = \left( {5.5\;{\text{min/ml}} \cdot {\text{VO}}_{2} + 1.76\;{\text{min/ml}} \cdot {\text{VCO}}_{2} - 26} \right)\;{\text{kcal/day}}$$

In this study, this is used as the reference method, against which other EE estimates are compared.

### Equations for estimation of EE

The equation for estimating EE based on VCO_2_ was constructed from Eq. 2, with VO_2_ substituted by:

3 $${\text{VO}}_{2} = {\text{VCO}}_{2} /{\text{RQ}}$$

This gives the modified Weir equation:

4 $${\text{EE}}\left( {{\text{VCO}}_{2} } \right) = \left( {\left( {5.5\;{\text{min/ml}} \cdot {\text{RQ}}^{ - 1} + 1.76\;{\text{min/ml}}} \right) \cdot {\text{VCO}}_{2} - 26} \right)\;{\text{kcal/day}}$$

VCO_2_ measurements used in the EE(IC) and EE(VCO_2_) estimations are both derived from the metabolic monitor. Differences between EE(IC) and EE(VCO_2_) must be either due to an incorrect assumption about RQ or due to variations in ventilation. Variations in ventilation will cause different variations in EE(IC) and EE(VCO_2_) because the time constant for VCO_2_ equilibration is much longer (10–20 min) [27, 28] than the time constant for VO_2_ equilibration (2–3 min) [29].

The accuracy of the EE(VCO_2_) estimates and that of some commonly used predictive equations (Table 1) were compared to EE(IC).

**Table 1:** Predictive equations for estimation of EE

Method		Equation
a	ACCP	The ACCP equation [9, 10, 30] using BM as the only variable
		EE(ACCP) = 25 kcal/kg/day · BM
b	Harris–Benedict	The Harris–Benedict equation from 1919 [11] multiplied by a SF
		Men: EE(HB) = (66.5 + 13.75 kg^−1^ · BM + 5.003 cm^−1^ · height − 6.775 year^−1^ · age) kcal/day · SF Women: EE(HB) = (655.1 + 9.563 kg^−1^ · BM + 1.85 cm^−1^ · height − 4.676 year^−1^ · age) kcal/day · SF
c	Harris–Benedict IBM	The Harris–Benedict equation with ideal body mass (IBM) multiplied by a SF
		Men: EE(HBI) = (66.5 + 13.75 kg^−1^ · IBM + 5.003 cm^−1^ · height − 6.775 year^−1^ · age) kcal/day · SF Women: EE(HBI) = (655.1 + 9.563 kg^−1^ · IBM + 1.85 cm^−1^ · height − 4.676 year^−1^ · age) kcal/day · SF
d	Mifflin St Jeor	The Mifflin St Jeor equation [12] multiplied by a SF
		Men: EE(MSJ) = (9.99 kg^−1^ · BM + 6.25 cm^−1^ · height − 4.92 year^−1^ · age + 166) kcal/day · SF Women: EE(MSJ) = (9.99 kg^−1^ · BM + 6.25 cm^−1^ · height − 4.92 year^−1^ · age − 161) kcal/day · SF
e	Penn State 1	The original Penn State equation from 1998 [13]
		EE(PS1) = 1.1 · HB + (32 min l^−1^ · MV + 140 °C^−1^ · *T*_Max_ − 5340) kcal/day
f	Penn State 2	Version 2 of the Penn State equation from 2003 [14]
		EE(PS2) = 0.85 · HB + (33 min l^−1^ · MV + 175 °C^−1^ · *T*_Max_ − 6433) kcal/day
g	Penn State 3	Version 3 of the Penn State equation from 2003 [14]
		EE(PS3) = 0.96 · MSJ + (31 min l^−1^ · MV + 167 °C^−1^ · *T*_Max_ − 6212) kcal/day

*ACCP* American College of Chest Physicians, *T*_*Max*_ maximum body temperature in 24 h (°C)

The cohort-specific value of SF for the Harris–Benedict equation (b, Table 1) was calculated using the following equation:

5 $${\text{SF}} = {\text{mean EE}}\left( {\text{IC}} \right)/{\text{mean EE}}\left( {\text{HB}} \right)$$

The SF for methods c and d (Table 1) were similarly determined using their respective mean EE. The result is that the mean EE for the 18 patients determined by each method equals the mean EE(IC) determined by Eq. 2 (the reference method).

The ideal body mass (IBM) was calculated from the Hamwi equations [31]:

6 $${\text{Men:}}\;{\text{IBM}} = 48.0\,{\text{kg}} + 2.7\,{\text{kg}} \cdot ({\text{height}}\,-\,1.524\,{\text{m}})/0.0254\,{\text{m}}$$

7 $${\text{Women:}}\;{\text{IBM}} = 45.5\,{\text{kg}} + 2.2\,{\text{kg}} \cdot ({\text{height}}\,{-}\,1.524\,{\text{m}})/0.0254\,{\text{m}}$$

### Sensitivity analysis of RQ

The practical use of VCO_2_-based calorimetry relies on a choice of RQ. A sensitivity study of the effect of the choice of RQ will be conducted. In six studies [14, 18, 32–36], the average reported cohort values for RQ ranged from 0.76 to 0.89. These minimum and maximum values and the extreme range of the physiological range (0.7–1.0) [23] will be used in the sensitivity analysis.

### Statistical analysis

#### Over-/underestimation

The bias of each method [the predictive equations and EE(VCO_2_)] was expressed by the difference in percent between mean EE for the method and mean EE(IC). The significance was tested by a two-tailed paired *t* test. The assumption of normal distribution of tested variables was assessed with the Shapiro–Wilk test.

#### Quality

The root mean square error (RMSE) was used to describe the quality of the predictions for each method. A comparison of EE(VCO_2_) and each predictive equation was performed by an *F* test over the prediction errors relative to EE(IC).

#### Accuracy

Per-patient EE estimates were defined as accurate if the estimate was within ±10 % of the IC measurement. The number of patients with accurate predictions was compared between EE(VCO_2_) and each predictive equation using Fisher’s exact test.

Significance level for all tests was *p* < 0.05. SPSS version 23 was used for statistical analyses.

### Qualitative analysis of dynamic errors

Both IC and VCO_2_-based calorimetry rely on the assumption that the rate of ventilated O_2_ and CO_2_ is reflecting the rate of O_2_ consumption and CO_2_ production, respectively. However, EE(IC) and EE(VCO_2_) calculated from instantaneous values of VO_2_ and VCO_2_ may be erroneous in situations where respiratory VO_2_ and VCO_2_ are not equal to the metabolically consumed or produced VO_2_ and VCO_2_, respectively. This may occur when the patient’s metabolism changes rapidly, or due to external changes to the patient’s ventilation. Patients were divided into a group with varying EE and a group with constant EE, according to the method described below. For a patient in each group, a descriptive analysis of the reasons for errors was performed by inspection of the 30-min recordings of MV, VCO_2_, VO_2_ and ET-CO_2_ and comparing these to the changes in EE(IC) and EE(VCO_2_).

### Quantitative analysis of dynamic errors

The effects of changes in ventilation were analyzed for both EE(IC) and EE(VCO_2_) to compare the two methods’ vulnerability to changes in ventilation. For each patient, the maximum deviation of EE from the mean EE was calculated for both EE(IC) and EE(VCO_2_). The effect of a 5-min moving average on the calculated EE was explored by comparing the maximum EE deviations from mean EE, for both EE(IC) and EE(VCO_2_), before and after its application.

### Method for assessing constancy of EE in individual patients

Each patient was analyzed for changes in EE during the 30-min recording period. The chosen marker for this analysis was VO_2_. EE(IC) is reliant on VCO_2_, and VCO_2_ takes 10–20 min to reach steady state following a change in ventilation pattern [27, 28], which implies that VCO_2_ and therefore also EE(IC) may not reflect the metabolically produced VCO_2_ for up to 20 min. Thus, both EE(IC) and VCO_2_ are unsuitable as markers for this analysis. VO_2_, however, reaches steady state after 2–3 min [29], implying that metabolic consumption of VO_2_ is equal to VO_2_ removed from inspired air. As this is a short period, compared with the 30-min recording period, VO_2_ was chosen as a metabolic marker for constant EE.

For each patient, the trend line for the VO_2_ recording was compared with the average VO_2_ over the recording period. If the difference between the trend line and the average was less than 10 % of the average VO_2_, the patient was considered to have constant EE throughout the recording period.

## Results

### Comparing estimates of energy expenditure

Eighteen patients (mean age 61 ± 17 years, five women) were included. Average VO_2_ for the 18 patients was 343 ± 77 ml/min and average VCO_2_ was 273 ± 63 ml/min, giving an average RQ of 0.81. The mean FiO_2_ was 42 % with no patient exceeding 50 %. All patients received intravenous glucose during the measurement period, and patients 1, 2, 3, 14, 17 and 18 received enteral nutrition. The mean RQ for the patients receiving enteral nutrition (0.86) was significantly higher (*p* < 0.05; *t* test, unpaired, two-tailed) than the mean RQ (0.79) for the patients not receiving enteral nutrition. Individual patient characteristics are given in Table 2.

**Table 2:** Patient data

Pt. no.	Age (years)	Height (cm)	Gender	BM (kg)	Meas. (h)	VO_2_ (ml/min)	VCO_2_ (ml/min)	RQ	MV (l/min)	*T*_Max_ (°C)	Vent. mode	Diagnosis	Apache2 score	Sedation
1	54	165	F	65	54	230	209	0.90	12.0	36.5	PS	S	18	No
2	55	165	M	60	44	189	159	0.85	7.4	34.1	VC	S	12	No
3	76	165	M	70	26	365	298	0.82	13.3	38.0	VC	T, ES	22	No
4	52	180	M	75	13	373	282	0.76	10.5	37.0	PS	S	17	Se
5	22	180	M	75	20	450	349	0.77	16.1	37.3	VC	S	14	An
6	60	179	M	73	1	294	228	0.77	6.9	35.1	VC	ES	6	Se
7	62	179	M	94	2	416	313	0.76	9.7	36.0	VC	SS	5	An
8	67	172	M	64	1	359	206	0.73	8.0	35.9	PS	SS	20	An
9	73	158	F	69	1	246	193	0.79	7.1	35.4	VC	SS	20	An
10	79	175	M	75	18	407	330	0.81	9.0	37.8	VC	ES	28	Se
11	56	173	M	105	1	416	371	0.89	12.0	35.5	VC	SS	16	An
12	81	155	F	84	1	248	223	0.90	6.4	36.5	VC	SS	12	An
13	82	180	M	100	19	389	310	0.80	12.4	37.1	PS	ES	17	Se
14	74	160	F	70	120	281	217	0.77	7.3	38.0	VC	ES	18	An
15	72	160	M	72	2	347	266	0.77	8.3	36.5	VC	SS	5	An
16	35	176	M	54	18	401	274	0.69	11.6	37.1	VC	ES	16	Se
17	55	170	M	75	72	351	335	0.96	12.0	37.8	VC	ES	29	Se
18	38	165	F	80	96	417	344	0.83	11.3	38.0	PS	T	11	An
Mean	61	170	–	76	28	343	273	0.81	10.1	36.6	–	–	15.9	–
SD	17	8.4	–	13	36	77	63	0.07	2.7	1.1	–	–	6.8	–

*Meas.* Time from ICU admittance to IC measurement, *VC* volume control, *PS* pressure support, *ES* emergency surgery, *SS* scheduled surgery, *S* sepsis/septic shock, *T* trauma, *No* no sedation, *Se* sedation, *An* anesthesia

**Fig. 1:**
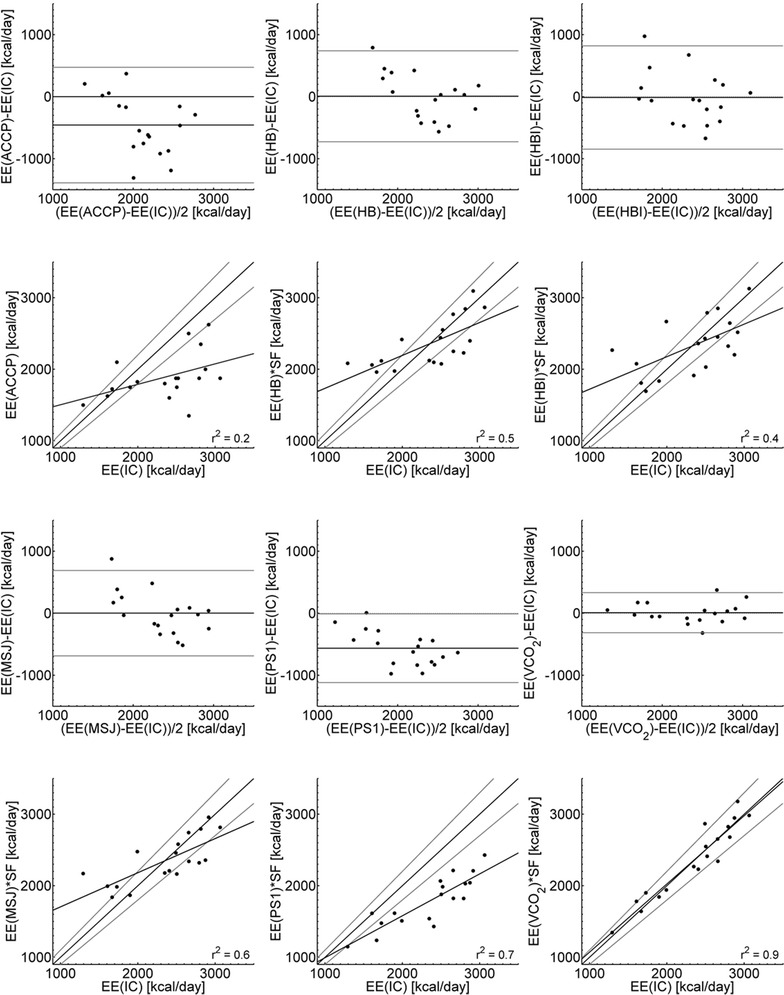
Bland–Altman plots and scatterplots for predictive equations and VCO_2_-based calorimetry compared with EE estimated using IC. Bland–Altman plots include 95 % limits of agreement. Scatterplots include lines marking *±*10 % of EE(IC) and linear regression lines with *r* ^2^ values

In summary, all predictive equations, a through g, largely over- and underestimated the reference EE value. The bias was the highest for the Penn State equations and the ACCP, while the ranges of estimation difference were largest for the ACCP, Harris–Benedict and Mifflin St Jeor equations (Table 3). The use of SF in the Harris–Benedict and Mifflin St Jeor equations resulted in these equations having a bias of 0 %; however, the quality of prediction was poor for all predictive equations, as reflected by a RMSE of 15 % or greater. Finally, the accuracy was also very poor for all predictive equations, with 50 % or less of patients having accurate EE estimates (Fig. 1).

**Table 3:** Comparison of EE estimates to IC including sensitivity of EE(VCO_2_) reliance on RQ

Equation	Mean EE (bias) (kcal/day)	SF	Range of estimation differences	RMSE of EE difference	# Of patients with accurate EE estimates (%)
ACCP	1889 (−20 %)*	NA	[−49 %; 22 %]	28 %^†^	6 (33 %)^‡^
Harris–Benedict	2347 (0 %)	1.55	[−20 %; 61 %]	16 %^†^	9 (50 %)^‡^
Harris–Benedict, IBM	2347 (0 %)	1.67	[−23 %; 76 %]	18 %^†^	8 (35 %)^‡^
Mifflin St Jeor	2347 (0 %)	1.59	[−18 %; 68 %]	15 %^†^	9 (50 %)^‡^
Penn State 1	1782 (−24 %)*	NA	[−41 %; 0 %]	27 %^†^	1 (6 %)^‡^
Penn State 2	1572 (−33 %)*	NA	[−49 %; −10 %]	35 %^†^	1 (6 %)^‡^
Penn State 3	1637 (−30 %)*	NA	[−43 %; −9 %]	32 %^†^	1 (6 %)^‡^
EE(VCO_2_) RQ = 0.81	2332 (−1 %)	NA	[−13 %; 14 %]	7 %	16 (89 %)
EE(IC)	2347 (0 %)	NA	–	–	–
Sensitivity analysis of RQ					
EE(VCO_2_) RQ = 0.70	2626 (12 %)*	NA	[−2 %; 30 %]	12 %	9 (50 %)^‡^
EE(VCO_2_) RQ = 0.76	2455 (5 %)*	NA	[−8 %; 20 %]	8 %	14 (78 %)
EE(VCO_2_) RQ = 0.85	2244 (−4 %)	NA	[−16 %; 10 %]	6 %	16 (89 %)
EE(VCO_2_) RQ = 0.89	2163 (−8 %)*	NA	[−19 %; 6 %]	10 %	10 (56 %)
EE(VCO_2_) RQ = 1.00	1976 (−16 %)*	NA	[−26 %; −3 %]	17 %	4 (22 %)^‡^

The bias in percent is relative to the mean EE(IC). The range of estimation differences is the maximum and minimum difference between the equations and individual mean EE(IC). The RMSE of EE difference is the root mean square error of EE difference between the equations and the IC measurements. Accurate EE estimates are defined as per-patient mean EE within ±10 % of EE(IC)

* Significantly different from mean EE(IC)

^†^Significantly greater variance than EE(VCO_2_) RQ = 0.81

^‡^Significantly different from EE(VCO_2_) RQ = 0.81

The EE(VCO_2_) was significantly better than the predictive equations with a low and acceptable bias. The mean EE(VCO_2_), with an RQ value of 0.81, was not significantly different from mean EE(IC), and the EE(VCO_2_) had a good quality of prediction with an RMSE of 7 %. The EE(VCO_2_) was accurate in 89 % of the patients, significantly better than the predictive equations. It also had the narrower range of estimation differences (Fig. 1).

### Sensitivity analysis of RQ

The sensitivity analysis showed that as long as the RQ is chosen within the published range of average cohort values, 0.76–0.89, the VCO_2_-based calorimetry performs better than the predictive equations.

### Analysis of dynamic errors in EE(IC) and EE(VCO_2_)

As explained earlier, changes in ventilation or rapid changes in patient metabolism can be causes of error in EE estimation. These errors will be described qualitatively and quantitatively.

#### Checking for constant EE

Out of the 18 patients, 17 were determined to have constant EE during the 30-min recording period, as the difference between VO_2_ trend line and mean was less than 10 %. For patients 1–17, the maximal deviation of the trend line from the mean was between 0.9 and 8 %. Only patient 18 had a major increase in metabolism with the VO_2_ trend line deviating 39 % from the mean.

#### Dynamic errors in patients with variable EE

**Fig. 2:**
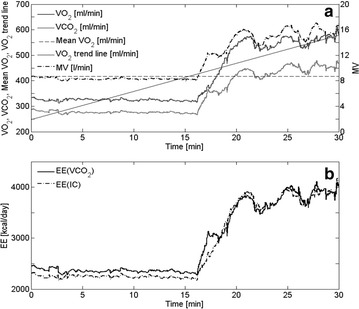
**a** Recorded VO_2_, VCO_2_ and MV from Patient 18. The mean and trend line of VO_2_ are also displayed. **b** EE(VCO_2_) and EE(IC) were calculated from recorded VO_2_ and VCO_2_

Figure 2a shows that for patient 18 the MV, VO_2_ and VCO_2_ are almost constant until 16 min where the patient apparently is aroused and all three parameters rise. VO_2_ increases by 78 % from 320 ml/min to about 570 ml/min and remains increased for over 10 min. If the increase had been due to the increased MV, without any increase in metabolism, then VO_2_ would have returned to its initial value of about 320 ml/min within 2–3 min. Since this does not happen, the prolonged increase in VO_2_ must therefore reflect an increase in metabolism.

Figure 2b shows that both EE(IC) and EE(VCO_2_), calculated from the recorded VO_2_ and VCO_2_, indicate increased EE, approximately to the same degree and simultaneously.

#### Dynamic errors in patients with constant EE

**Fig. 3:**
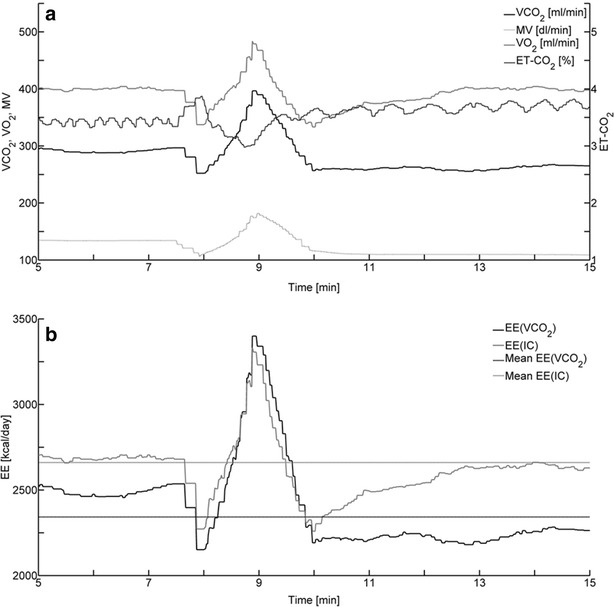
**a** Recorded values from Patient 16 of VCO_2_, ET-CO_2_, VO_2_ and MV. **b** EE(VCO_2_) and EE(IC) calculated from recorded VO_2_ and VCO_2_, including means of EE(VCO_2_) and EE(IC)

Most of the 17 patients with constant metabolism had one or more changes of ventilation. Patient 16, whose VO_2_ trend line deviated 2.7 % from the mean VO_2_, will be used as an example. The patient, who was volume controlled, had two changes in ventilation (Fig. 3a): a 3-min period of unstable MV from 7.5 to 10.5 min and a sustained reduction in MV from 10 min until the end of the recording.

During the unstable period, MV reached a peak value which is 36 % higher than the steady-state value up to 7.5 min. This gave rise to increases in VO_2_ and VCO_2_ of 22 and 34 %, respectively, which were mirrored as increases in EE(IC) and EE(VCO_2_) of about the same size, 24 and 35 %, respectively (Fig. 3b).

The second change in ventilation was a sustained reduction in MV at 10.5 min from 13.5 to 11.7 l/min. As a result of the reduced ventilation, ET-CO_2_ rises, but does not quite reach steady state, because of its 10- to 20-min equilibration time constant. For the same reason, VCO_2_ remains low, but rises slowly from 10.5 min and on. In contrast to VCO_2_, VO_2_ equilibrates within a few minutes and returns to its original value of about 400 ml/min, indicating that there is no reason to suspect that the patient’s EE changes during the 10-min period shown in Fig. 3. Therefore, the fluctuations of EE(IC) and EE(VCO_2_) must be ascribed to the fluctuations of MV.

The changes in VO_2_ and VCO_2_ are reflected in the changes in EE(IC) and EE(VCO_2_) (Fig. 3b). At 12.5 min, EE(IC) has almost recovered and reached its original value of 2720 kcal/day. EE(VCO_2_) remains low, although it increases slowly.

The conclusion on this qualitative analysis is that rapid changes in MV (a rise or fall with a duration of less than 1 min) are reflected about equally in EE(IC) and EE(VCO_2_), that during maintained changes in MV, EE(IC) largely recovers within a few minutes and that EE(VCO_2_) will take 10–20 min or more to recover.

#### Quantitative analysis of dynamic errors

The effect of changes in ventilation is given in Table 4 for each of the 17 patients with stable ventilation. It can be seen that both EE(IC) and EE(VCO_2_) are vulnerable to changes in ventilation. EE(IC) has up to 42 % deviation (Patient 8), and EE(VCO_2_) has up to 46 % (Patient 16) deviation. EE(IC) and EE(VCO_2_) are about equally vulnerable with no significant differences (*t* test) between the mean of the max values for the two methods. In clinical practice, this implies that an instantaneous reading of EE(IC) and EE(VCO_2_) cannot safely be used to assess EE.

**Table 4:** Maximal deviations from mean EE and from a mean of EE after the inclusion of a 5-min running average of EE, for both EE(IC) and EE(VCO_2_)

	Max EE(IC) versus EE(IC) (%)	Max EE(IC) versus 5-min EE(IC) (%)	Max EE(VCO_2_) versus EE(VCO_2_) (%)	Max EE(VCO_2_) versus 5-min EE(VCO_2_) (%)
1	−7	−2	−11	−3
2	22	−12	−20	−13
3	12	−6	14	−5
4	−20	4	−21	8
5	−4	1	−5	1
6	−4	−3	−2	−1
7	15	5	−7	4
8	42	11	−38	11
9	11	9	8	−6
10	31	18	−24	14
11	−3	−1	2	1
12	−6	−3	−3	2
13	20	4	5	2
14	−7	−2	−6	−3
15	−9	13	−9	11
16	−28	9	46	12
17	11	4	17	7
Mean (±SD)	4.4 (±18.3)	2.8 (±7.5)	−3.2 (±18.9)	2.5 (±7.3)
RMS	18	8	19	8

Deviations are expressed as a percentage of the mean EE

Applying a 5-min moving average to the calculated EE(IC) reduced the max deviation to 18 % (Table 4, column 3, Patient 10) and the SD of the mean to 7.5 %. For EE(VCO_2_), the max deviation was reduced to 14 % (Table 4, column 5, patient 10) and the SD of the mean to 7.3 %.

This means that the introduction of a 5-min running average reduced the dynamic error of the EE(VCO_2_) to a size comparable to the RMSE of EE difference (Table 3).

## Discussion

The goal of this study was to investigate the accuracy of EE estimates by predictive equations and by VCO_2_-based calorimetry in a small cohort of critically ill patients, most of them soon after admission to the ICU. The results corroborate the previously reported [6, 15] inaccuracy of predictive equations for EE. Tatucu-Babet et al. [6] found underestimations of EE up to 41 % and overestimations up to 66 %, which is similar to the results in this study. In our study, even the best of the equations, the Mifflin St Jeor equation, was accurate only in 50 % of the patients.

The two predictive equations with the best performance in our study were Mifflin St Jeor and Harris–Benedict. Both of these equations have the methodological problem that they require a SF to account for the increased metabolism following an insult. The SFs giving the best fit to our data were 1.59 and 1.55 for the two equations, respectively. Published mean values for SF for different cohorts range from 1.13 to 1.6 [6], and our cohort values for SF thus fall close to the upper end of the published range. This may partially be due to statistical fluctuations due to our small number of patients, but in general the large range of reported SF implies that SF used must be adapted to the cohort of patients. An additional problem is that EE, and thus SF, tends to increase for the first 9–11 days [16, 17] after the insult that led to the admission to the ICU.

In our small sample of ICU patients, VCO_2_-based calorimetry estimated EE accurately in most patients (89 %), even in cases where ventilation was changing during the recording period. VCO_2_-based calorimetry performed significantly better than all predictive equations in agreement with earlier findings both in adults and in children [21, 22].

However, VCO_2_-based calorimetry has two methodological challenges. The first is that the method requires a choice of RQ to be made, and the second is that the accuracy of the estimation is affected by instant variability in measurements of MV and VCO_2_.

RQ was fitted to our cohort by choosing the average value of RQ for the cohort in the calculation of EE(VCO_2_). In practice, the value of RQ for the cohort will not be available, and the robustness of VCO_2_-based calorimetry was explored by a sensitivity analysis. The analysis showed that for any choice of RQ within the published range of cohort values for RQ (0.76–0.89) [14, 18, 32–36], the EE(VCO_2_) equation performed significantly better than the predictive equations. The results of the sensitivity analysis show that as long as the RQ value chosen by the clinician is within the published range of values, the estimation of EE will be better compared with predictive equations.

The use of nutritional RQ has been explored both in children [21] and in adults [22], and both failed to provide evidence that EE estimates are improved by using nutritional RQ. In children [21], the nutritional RQ gave poorer estimates than the mean RQ for the cohort. For the patients in our cohort, a nutrition-based RQ would have given poorer accuracy, as evidenced by the observation that contrary to expectations the patients receiving only glucose had a significantly lower RQ than the patients also receiving enteral nutrition. An explanation of the failure of nutritional RQ to improve EE estimates may be due to the mobilization of the patient’s own energy stores in the early catabolic phase of critical illness, where plasma concentrations of glucose, fatty acids and amino acids are strongly increased, thus weakening the link between nutrition and metabolism [16, 17].

If a suggestion is to be made on a choice of RQ for VCO_2_-based calorimetry, the authors suggest 0.85 as this number is in the middle of the physiological range (0.7–1.0); is within the published range of cohort values for RQ (0.76–0.89); gives an acceptable −4 % mean EE difference from IC; gives the smallest RMSE (6 %); and is the highest number of accurate EE estimates in this cohort.

The second methodological problem with VCO_2_-based calorimetry is that EE(VCO_2_) is inaccurate during and immediately after changes in MV. A qualitative analysis showed that instant values of EE(IC) were almost as vulnerable to fluctuations in MV as EE(VCO_2_) with fluctuations about the same size as the fluctuations in MV. This behavior is compatible with the 10- to 20-min time constant for VCO_2_ equilibration, supported by both mathematical models of VCO_2_ storage and transport [28] and experimental data [27]. The problems arising from fluctuations in MV and thus VCO_2_ and VO_2_ are less pronounced when using IC as the equilibration time for VO_2_ is 2–3 min, and as can be seen from Eq. (2), VO_2_ has the larger influence on the EE estimation. Smoothing EE(VCO_2_) and EE(IC) with a 5-min running average reduced the sensitivity to fluctuations in MV and reduced the RMSE of the maximum deviations from 19 and 18 %, respectively, to 8 % for both of them. Although a 5-min average thus substantially reduced the variability of EE(VCO_2_) and EE(IC), it is still advisable to avoid using measurements taken during fluctuations or up to 20 min after changes in MV to allow for equilibration of VCO_2_. Alternatively 24-h measurements of VCO_2_ could be used in the VCO_2_-based calorimetry. Using the mean 24-h value has benefits over a 30-min measurement period as the influence of fluctuations from hypo- or hyperventilation on EE(VCO_2_) and EE(IC) is eliminated, reducing the discrepancy between metabolic production and pulmonary uptake or excretion.

The widespread availability and relatively low cost of capnometers, and software to analyze VCO_2_ from CO_2_ concentrations and expiratory volume, may make VCO_2_-based calorimetry a simple and accurate method for determination of EE in critically ill patients, whenever needed. Production of the most extensively used IC system (Deltatrac Metabolic Monitor) has been discontinued, and newer available IC systems give conflicting EE estimates [37]. Thus, in the absence of other devices validated for use in the ICU, use of CO_2_-based calorimetry can represent a useful alternative for the determination of EE.

## Abbreviations

EE: energy expenditure
VO_2_: measured oxygen consumption
VCO_2_: measured carbon dioxide production
IC: indirect calorimetry
BM: body mass
MV: minute volume
SF: stress factor
RQ: respiratory quotient
N: nitrogen metabolism
